# Perspectives of health workers on malaria case referral among pregnant women attending antenatal care in Savelugu Municipality, Ghana: A qualitative descriptive study

**DOI:** 10.1371/journal.pone.0319567

**Published:** 2025-03-18

**Authors:** Martin Nyaaba Adokiya

**Affiliations:** Department of Epidemiology, Biostatistics and Disease Control, School of Public Health, University for Development Studies, Tamale, Ghana; Universite de Parakou, BENIN

## Abstract

**Background:**

Malaria remains endemic in Africa, with pregnant women exposed to the dangers of complications, which require emergency referral for lifesaving interventions. This study explored the perspectives of health workers on malaria case referral among pregnant women attending antenatal care (ANC) at health facilities in Savelugu Municipality, northern Ghana.

**Methods:**

The study employed a qualitative descriptive design with a constructivist grounded theory perspective. A semi-structured interview guide was used to collect information from thirteen health workers on face-to-face. The participants were purposefully sampled and interviewed. The data were transcribed verbatim. Content analysis was performed and the results were presented as themes and sub-themes.

**Results:**

After the analysis, two main themes and six sub-themes emerged from the data: a robust referral system (patient awareness and consent, documentation, referral coordination, and post-referral feedback) and a referral mechanism (ambulance/nurse accompaniment, and transportation shortfalls/patients left to their fate). Participants described the referral system as robust but reported transport challenges as the main obstacle to its operation. The study also revealed that health workers do not accompany all referral cases.

**Conclusion:**

A referral system exists in the Savelugu Municipality with transportation lapses causing delays. Health workers often failed to accompany referred cases to the referral facilities. Though an inter-sectoral approach is needed to tackle the referral transport challenges, facility managers need to ensure that all referred cases are accompanied by a designated health worker.

## Background

Despite global efforts to end malaria, sub-Saharan Africa (SSA) remains endemic to the infection, with over 70% of all global cases [1]. Malaria in pregnancy poses a significant risk to both mothers and their unborn babies and usually leads to poor birth outcomes [2–4]. It is estimated that malaria directly accounts for over 25% of all pregnancy related complications in SSA [5]. Thus, malaria in pregnancy requires robust management efforts including prompt referral for lifesaving interventions. Existing preventive interventions including insecticide treated nets (ITN) and intermittent preventive therapy (IPT) continue to experience low success rates [6]. For instance, high rates of malaria cases recorded among pregnant women in Zambia were attributed to limited successes of past interventions [7]. Similarly in Ghana, preventive measures have been reported as sub-optimal and malaria was implicated for many obstetric referrals [8]. In the Northern Region of Ghana, pre-term delivery and low birth weight have been cited as some malaria related complications among pregnant women [9].

People living in malaria endemic areas build immunity to the parasite overtime [10]. However, pregnancy presents additional risks which makes women prone to acquiring severe infections, and placental sequestration of malaria parasites mostly leads to poor birth outcomes including low birth weight, premature birth, intrauterine death, congenital malaria among others [11]. This is more so in high transmission areas where placental malaria is prevalent among primigravidae [12]. While there are renewed calls to intensify awareness creation, increasing access and utilization of preventive interventions, effective management of malaria cases including prompt referral is paramount [13]. Pregnant women who develop severe malaria need to be referred to well-resourced facilities to avert complications [5,14]. However, various healthcare systems have different approaches for referrals. The World Health Organization’s (WHO) standard for maternal care stipulates that pregnant women must be thoroughly assessed, treated with available resources, or referred promptly [15]. Additionally, appropriate referral of maternal cases is indicative of a robust healthcare system [16].

The referral system involves steps and processes around identification, consent, documentation, communication, and referral feedback [17,18]. These processes, among other things, should help remove delays in accessing emergency maternal care. For instance, effective inter-facility communication ensures that patients receive prompt care, as referred cases do not join queues at referral facilities [19]. Contrary to expectations, significant delays in reaching and receiving treatment have contributed to the high burden of preventable maternal deaths observed in Africa [20]. Thus, the referral system is not an end itself, but the means to reaching timely lifesaving interventions at adequately resourced health facilities. Though numerous referral interventions exist, recent reviews highlighted the paucity of data on the effective implementation of such interventions in SSA [17]. Additionally, the referral system needs to meet certain requirements to remain viable. For an efficient referral system, patients must be transported swiftly to the referral center, which makes an active transport system pivotal to the referral process. Yet, transportation has always been a major challenge to the smooth functioning of the referral system in developing countries [15,16,21]. Pregnant women in rural settings are particularly disadvantaged, as poor road network makes such communities inaccessible to motorists [21].

In Ghana, the National Referral Policy Guideline was developed in 2012 in response to the growing need for emergency care [22]. It provides technical guidelines for managing the referral system, but with a strong focus on strengthening primary healthcare. The referral system operating within the Ghanaian healthcare system begins at the community level (via Community-based Health Planning and Services, CHPS) and proceeds through the sub-district level to the district hospitals [19,22]. The district hospitals in turn refer cases to the regional or teaching hospitals. Similar to many African countries, Ghana has its own referral system challenges. A survey on CHPS operations found that community health officers (CHOs) had challenges implementing appropriate referral interventions for malaria cases [13]. It has also been reported that patients usually bypass the gatekeeper (community level) healthcare system and move to higher levels, thereby increasing caseloads at designated referral facilities [22]. Additionally, breaks in inter-facility communication, poor documentation, transport challenges, and inadequate referral feedback have been identified [21]. Referral feedback is particularly important as it completes the referral process and informs referring facilities of the outcome of the referred case [22]. Inability to give referral feedback may imply a break in the continuity of patient care.

Pregnancy-related complications are often unpredictable thus, the need for emergency referrals [19]. But with many infractions within the referral system, there is a fertile ground for poor maternal health outcomes. Considering that Savelugu Municipality is composed predominantly of under-resourced lower-level facilities (CHPS and health centers), a recent study reported suboptimal practices relating to the management of malaria in pregnant women [23]. With this evidence, there arise concerns about how pregnant women with severe malaria are referred as part of the protocol for comprehensive maternal care. Evaluating the referral system may uncover lapses and help provide broader insights to guide policy changes. Therefore, this study explored the perspectives of health workers on malaria case referral among pregnant women attending antenatal care at health facilities in Savelugu Municipality of northern Ghana.

## Materials and methods

### Study design

The study employed a qualitative descriptive design with a constructivist grounded theory perspective [24,25]. In-depth qualitative interviews were conducted among thirteen health workers on face-to-face. The participants provided ANC services, including screening for malaria among pregnant women following recommended protocols [14]. Participants’ perspectives on malaria case referral among pregnant women attending ANC at health facilities were explored. The study was conducted in line with the qualitative descriptive approach as it is flexible and also enabled the researcher to provide a straightforward description of participants experiences and perceptions on the research question [24]. The data was collected from January 28, 2020 to February 9, 2020.

### Study setting and participants

The study was conducted in the Savelugu Municipality of Northern Ghana. Specifically, it was conducted in four health facilities within the Municipality, namely the Savelugu Reproductive and Child Health Center (primary healthcare and referring facility), the Moglaa Health Center (primary healthcare and referring facility), the Savelugu Municipal Hospital (district hospital and main referral facility in the municipality), and a Private Laboratory (runs routine laboratory test including microscopy for malaria parasites). The participants were purposefully selected for the study based on their roles and experiences in the provision of maternal and child healthcare services during pregnancy as well as their role in diagnosing malaria among pregnant women. The participants included medical officers, midwives, nurses, and laboratory professionals (Table 1).

**Table 1:** Sociodemographic characteristics of study participants.

S/N	Gender	Role	Work experience (years)
1	Female	Principal Midwifery Officer	7
2	Female	Midwifery Officer	6
3	Female	Midwifery Officer	2
4	Female	Senior Staff Midwife	4
5	Male	Senior Staff Nurse	5
6	Male	Senior Staff Nurse	4
7	Female	Staff Nurse	4/12
8	Female	Enrolled Nurse	6
9	Female	Enrolled Nurse	2/12
10	Female	Community Health Nurse	1
11	Male	Laboratory Technician	6
12	Male	Principal Biomedical Scientist	9
13	Male	Medical Officer	1

### Inclusion and exclusion criteria

This study employed purposive sampling technique to recruit health workers who were willing to grant an interview and give rich quality data to answer the research question. Thus, decision to recruit was based on availability and willingness, as the most experienced persons available at each health facility were recruited. Another prerequisite was for participants to be engaged in providing direct care to pregnant women, or have a functional role in the referral process. However, health workers who lacked experience in the referral process or do not provide direct care to pregnant women were exempted from the study as per the national guideline for malaria management [14].

### Data collection

Prior to commencing the data collection, a semi-structured interview guide was developed based on prior literature review. The tool was then shared for experts’ input and refined, before rolling out the study. Additionally, the interview guide was pilot tested on three health workers from two facilities in the municipality to check for coherence and relevance of the questions (but whose data were not included in the final analysis). Before enrolling participants, the nature of the study was explained, and they decided the time and place for the interviews. They were also permitted to ask questions and assured of the confidentiality of the data. Those who agreed to participate in the study signed a written consent form individually.

The participant interviews started with general questions about experiences in healthcare, followed by questions on referrals and malaria treatment among pregnant women. Follow-up questions such as ‘What does that imply?’ or ‘Can you give an example?’ were asked to clarify the perspectives of participants. Each interview lasted about forty-five minutes (average of 45 minutes with a standard deviation of 10 minutes). The interviews were discontinued when the data reached saturation, at which point no new codes could be formed in subsequent interviews [26]. Data saturation was attained after the 11^th^ interview with the gradual generation of a potential list of codes. However, two additional interviews were conducted to ensure that no new codes emerged from the data. All the interviews were conducted face-to-face in English Language and audio recorded with the consent of the participants.

### Data analysis

The study applied qualitative content analysis to analyze the data through an iterative inductive process [27]. The audio recordings were transcribed verbatim. The transcripts were read repeatedly to become familiar with the information. Then, the texts were read word-by-word, and initial codes were identified. These included recurrent phrases and underlying concept of participants’ perceptions. The coding process was then conducted line-by-line. The codes were organized into related categories, forming broad themes that reflected patterns in the data and were relevant to the study objective. The themes were reviewed and assessed to determine if they reflected the context of the source data. The investigator then redefined and organized the themes into related, meaningful wholeness, which led to the formation of main themes and sub-themes. The analysis process was discussed with experts in the qualitative research field.

### Rigor of findings

To enhance the rigor of the study findings, the Lincoln and Guba’s criteria, which include credibility, dependability, confirmability, transferability, and authenticity was adopted [28,29]. Credibility was attained through prolonged engagement with the data, member checking, and peer debriefing. Additionally, two experts in qualitative research were invited to validate the codes or data analysis process as a way of ensuring peer debriefing. For the dependability of the findings, the coding process was described in details, while the participants’ perspectives were captured as thick and rich text [30]. The codes were also shared with the participants for verification, and when necessary, corrections were made to the initial codes to reflect their perspectives as a way of ensuring member checking. For transferability of the findings, the investigator ensured that the research method and processes (participant selection, data collection/analysis, and report writing) were related to the study objective [29].

### Ethics consideration

This study was approved by the Ghana Health Service Ethics Review Committee (with registration number GHS-ERC 017/06/19). We received permission from the Northern Regional Health Directorate of Ghana Health Service (GHS) to conduct the study. In addition, we also received permission from the Savelugu Municipal Health Directorate of GHS. The participants signed individual written informed consent forms and were assured that they could withdraw from the study without consequences.

## Results

In all, thirteen interviews were conducted among different categories of health workers, comprising six nurses (who conduct routine assessment, care for and make suggestions for referrals); four midwives (deliver maternal care, assess and make referrals); two laboratory workers (conduct laboratory test on blood samples, communicate results and form part of referral coordinating team); and one medical officer (in charge of diagnosing and given various treatment options including ordering for referrals). Five of the participants were males and eight were females. Averagely, the participants had service experience of 3.6 years (Table 1).

### Themes and sub-themes

In this study, health workers’ perspectives on malaria case referral were explored. Two main themes and six sub-themes emerged from the data (Table 2).

**Table 2:** Main themes and sub-themes of the study.

Theme	Sub-theme
The robust referral system	Patient awareness or consent
	Documentation
	Referral coordination
	Post referral feedback
Referral mechanism	Ambulance/nurse accompaniment
	Transportation shortfalls/Patient left to her fate

### The robust referral system

Exploring the perspectives of participants revealed that the existing referral system is robust and efficient in the Savelugu Municipality. Their insights are summarized into four sub-themes, including patient awareness and consent, documentation, referral coordination and referral feedback. Accordingly, these arrangements facilitate the management of malaria in pregnancy without significant delays. Referred cases quickly move through the various healthcare delivery systems, from less to more resourced facilities, for timely and lifesaving interventions

### Patient awareness/consent

According to the participants, the first stage of the referral process is informing the patient and seeking her consent. They explained that referral becomes necessary when the referring health facility feels inadequate to handle the condition of the pregnant woman or client. The rationale for the referral is usually explained to the pregnant woman and her significant others to gain their cooperation.

*“The client is informed that the situation cannot be handled here. We need to get to a bigger health facility where they have specialized people to take care of the person”* **(Enrolled Nurse #9)**.

### Documentation

From the health workers’ perspectives, documentation is another crucial component of the referral process. They emphasized the need to include the patient’s name, contact details, complaints, pre-referral treatment, and any other pertinent issue regarding the referral. They added that proper documentation helps track the patient’s progress in the referral process.

*“You bring the folder and enter the person’s name, gestational age, and the person’s age. With the attendance book, we are able to tell if the person was registered here or not* (At this health facility or a different one)*…. We write every piece of information about the person and why we are referring her”* **(Enrolled Nurse #8)**.

Another participant corroborated this, and added that the referral form is filled in duplicate with a copy left for the referring facility:

“*A referral form is filled out with the client’s details, complaints and the treatment given at this stage. The client is then accompanied by relatives, but if her condition is severe, the ambulance is used. And a duplicate of the filled-out referral form is kept in the health facility*” (E**nrolled nurse #9).**

### Referral coordination

Robust coordination is at the heart of the referral system. The health workers indicated that a well-coordinated referral system exists in the Savelugu Municipality. They added that the Municipality has a common platform (WhatsApp) for coordinating all referral cases. Besides, the participants revealed they still follow-up with phone calls on all referred cases. Consequently, the health workers constantly communicate and are able to track the progress of a patient referred from one level of the healthcare system to another*.*

*“We have a system for referrals here. They will call if they want to refer to Tamale. They will call Tamale Teaching Hospital that we are bringing a case for them to know that they are sending a case. Then, they will refer, and the pregnant woman will go with the referral note to the next level”* **(Principal Midwifery Officer #1).**

“*The Savelugu Municipality has a referral WhatsApp platform for pregnant women known as the labor room platform, with all health workers (*midwives, medical assistants, doctors etc*.) on board. Anytime there is a case, a picture of the client’s referral form is posted on the platform”* (**Staff Nurse #7**).

The participants further elaborated that there is a referral coordinator in the Savelugu Municipal Hospital. With this arrangement, clinicians concentrate on their work while the coordinator performs the administrative aspect of the referral process. The coordinator calls the next- level health facility and arranges for transportation as well.

“*We have referral coordinators in every unit from here and they also have their coordinators at Tamale Teaching Hospital. We have set up coordinators who will communicate, so I do not necessarily have to call. Once I make my intention clear to the ward, I can just call our coordinator and he will call the ward coordinator that the patient is going. Then they will make arrangements to receive the patient, and they will give us feedback”* **(Medical Officer #13)**.

### Post- referral feedback

The health workers also revealed that sending a patient out of their facilities is not the end of the referral process. From their perspective, they also receive feedback on the progress of the referred case. This is usually a mutual process; either the referral or referring facility can call and give/get an update on the condition of the case.

*“And also because of the feedback system, so when you send the person, the ward receives and gives feedback. And then follow up. Once you receive the client, your management has to update the senior colleagues on the page on what you are doing and the outcomes of your interventions. This is done until the patient is stable”* **(Medical Officer #13)**.

### Referral mechanism

The participants also shared experiences regarding the medium through which referred patients are moved from one level of the healthcare system to another. From their perspectives, two sub-themes emerged: ambulance/nurse accompaniment and transportation shortfalls/patient left to her fate. It emerged that the availability of ambulance for clients depended on the facility involved. Clients from the district hospital enjoyed ambulance services whereas their counterparts at the lower-level facilities do not have easy access to vehicles. They mostly relied on motor bikes from relatives, community members of health workers. Regardless of which ever approach, it comes with a cost for the pregnant woman and or family. These dynamics also determined if a patient is accompanied to the next level facility by a nurse or not. The participants revealed that they are bond to accompany a patient when there is an ambulance to convey her, otherwise the patient would have to go without a nurse.

### Ambulance/nurse accompaniment

According to the participants, the referral system also includes transport services as a component. They reiterated that transportation is arranged to convey the pregnant woman to the referral facility. The participants further explained that some facilities have ambulances for this purpose. In most cases, a nurse or midwife accompanies the pregnant woman to ensure her safety and manage any unexpected complications that may arise while on the journey.

*“We take another assistant nurse to help the patient to the next facility because we can’t just leave the patient alone. We make sure that a nurse goes with the relatives of the patient. So, when they reach there, the nurse will explain why he/she is asked to bring the client”* **(Enrolled Nurse #8)**.

This was buttressed by another participants:

*“We have two ambulances in this facility that usually convey our patients. As I mentioned earlier, the referral coordinator also makes arrangements for transportation. He engages the ambulance crew and makes sure everything is set before the patient is moved out of the ward”* **(Medical Officer #13)**.

The health workers also revealed that patients may have to pay for ambulance transport. However, the patient could make payment either before or after the referral.

*“…They are transported by an ambulance. Arrangements will be made, and the relatives will be required to pay some fees…. Sometimes too, some people have financial challenges, but the hospital still transports them, so later they engage the patient’s relatives and they are able to settle the bill”* **(Principal Biomedical Scientist #12)**.

### Transportation shortfalls/patient left to her fate

The health workers also reported that there were moments when they experienced challenges transporting referred patients to the next level facility. They sometimes offered their own motorbikes or relied on community members to convey ailing pregnant women to higher-level facilities. Without ambulances, participants found it difficult to accompany the referrals.

“*When they come and it is beyond us, we quickly give our pre-referral treatment and then we fill out the referral form. Normally, we do not go with them, unless the person is unstable. We do not have an ambulance at this health facility. So, when the person is severely ill, we support them with our own means, our motorbikes, sometimes we call their relatives or any community member if they can provide support”* **(Senior Staff Nurse #5)**.

## Discussion

This study explored the perspectives of health workers on malaria case referral among pregnant women attending antenatal care at health facilities within Savelugu Municipality, Ghana. Two main themes and six subthemes emerged from the data: a robust referral system (patient awareness/consent, documentation, coordination and referral feedback) and the referral mechanism (ambulance/nurse accompaniment and transportation shortfalls/patient left to her fate).

From the results, a robust referral system exists in the Savelugu Municipality. In the current study, the health workers described the referral system as efficient due to the arrangements that revolve around patient awareness/consent, documentation, coordination and referral feedback. With these arrangements, health workers are able to refer and track the progress of a referred case at any level of the healthcare system. This finding is consistent with guidelines stipulated in the Referral Policy of Ghana [22]. The policy stipulates the requirements for referring patients and emphasizes the need for effective coordination, documentation, and communication between the various levels of healthcare organizations in the country. A recent review also reported adequate evidence of referral identification, documentation, and communication interventions within health facilities in sub-Saharan Africa [17]. However, individual studies presented contrasting findings. In Ghana and Kenya, poor communication between health facilities was cited as a major barrier to the referral system [21,31]. Inter-facility communication is crucial for prompt delivery of care. It prevents delays in patient care as referred cases do not have to queue at the referral facility [19]. Interventions to maintain a continuous flow of information between referring and referral facilities may contribute to quality patient care delivery.

While mobile phones were mostly used for communication (through short message service- SMS alerts) in previous studies [17], social media (WhatsApp) usage was predominantly reported in the current study. Recent studies have reported that WhatsApp is becoming a viable channel for coordinating obstetric referrals both in southern Ghana [32,33] and elsewhere in Africa [34]. In this study, the use of the WhatsApp massaging application was reported to enhance coordination of the referral process. It created a platform for varying expert opinions (from both facilities) on cases being referred. Consequently, both referring (sending) and referral facilities (receiving) were adequately informed and able to track the progress of a referral case.

In the current study, referral feedback was adequately implemented. Referral feedback is a fundamental requirement as it completes the referral process and enhances continuity of care at referring facilities [22]. Previous studies have had inconsistent results with this requirement [17,21]. Consequently, patients may be lost to follow-up for care or fail to adhere to their treatment regimen [35]. In view of the challenges confronting many referral systems, the current finding may be of interest to health systems within and outside the Ghanaian context. Strategies identified in this study may be adopted for smooth and efficient referral of patients in other jurisdictions.

In the current study, findings also revealed that health workers in some facilities do not accompany patients on referral. Patients were left to their fate and had to find their way to the referral facilities. Previous studies conducted in Ghana also reported similar lapses regarding patients referred from one level of the healthcare system to the other [19,21]. Similarly, findings from a recent review reported that most referral cases were not accompanied by health workers in SSA [16]. This finding contravenes provisions of the national policy on patient referrals, which mandates health workers to accompany all referral cases [22]. When patients were unaccompanied in similar situation, they either misplaced or failed to release their referral forms to the referral facility with the aim of getting a fresh opinion on their condition [36]. This disrupted the smooth transfer of care continuity due to a break in communication between referring and referral facilities. Consequently, patients may be exposed to repeated and needless assessment, laboratory testing, treatment delays, as well as incurring additional healthcare costs. Additionally, complications may arise during patient transportation. Therefore, it is a fundamental requirement for health workers to accompany and manage such complications when moving patients from one facility to another [14,22]. This is even more applicable for pregnant women with malaria as a severe form of the disease can progress into complications within short duration [14]. While it is imperative for government to implement policies targeting adequate staffing, health facility managers should adhere to the national policy guidelines on ensuring that nurses/midwives accompany patients (particularly pregnant women with malaria) during referral.

Regardless of the apparent functional referral system that exists in the Savelugu Municipality, most facilities have transport challenges. Participants in the current study acknowledged that the referral system is robust but identified transportation shortfall as the major hindrance to its function. The facilities (communities) located at the periphery of the municipality were the most effected due to poor road network and unavailability of vehicles. This problem is not peculiar to Savelugu Municipality as previous studies reported poor transportation services as one of the causes of delays in accessing maternal care in Africa [21,37,38]. In the absence of ambulances, private commercial vehicles became the only alternative for transporting referral cases [19,39]. This is also consistent with the current finding that motorbikes were used to transport ailing pregnant women in desperate situations. Depending on commercial transportation has both financial and safety implications. It delays the referral process and undermines maternal care delivery [40]. In most rural Ghanaian communities, poor road network accounts for transportation challenges [21]. Hence, building a resilient referral system demands more than policy documents. The provision of basic infrastructure is pre-eminent. An inter-sectorial approach through the collaboration of finance, transport, and health ministries could increase the sensitivity of the government to maternal health needs [15]*.* Therefore, there is a need to ensure the provision of the needed infrastructure and logistics in terms of building good roads and procuring more ambulances. The government should set this as a priority in its agenda for a healthy population for national development.

However, the referral system may be counter intuitive if it is not properly managed. Though it helps patients access appropriate lifesaving interventions, there are concerns about abuse, which could put undue pressure (due to increased caseloads) on referral facilities. Improperly referring cases to under- resourced facilities may imply ‘referring mortalities’ [17]. Additionally, frequent referrals to higher level health facilities may erode confidence in the gatekeeper’s healthcare system, which forms the basis of healthcare delivery in Ghana [21]. Appropriately referring patients may reduce overreliance on and/or burden on higher-level facilities. Hence, strengthening the primary healthcare system would empower them to handle most cases, which could lead to fewer referrals as suggested in previous studies [21,41]. The current study could not ascertain the technical capacity of the healthcare system (particularly, the lower-level facilities) in identifying cases for appropriate referral in the Savelugu Municipality. Therefore, future research efforts may be geared toward assessing the capacity of health facilities to handle emergencies and complications, as well as the ability of health workers to correctly identify and refer cases beyond their strength.

### Strengths and limitation of the study

The current study has some strengths. Firstly, the adoption of a constructivist grounded theory methodology allowed a better exploration and understanding of health workers’ perspective on referral of pregnant women with malaria and inherent challenges. Secondly, the design was flexible and thus, allowed a purposeful selection of the study participants. Additionally, it is the first study to explore health workers’ experiences on malaria case referral among pregnant women attending antenatal care at health facilities in Savelugu Municipality.

This study also has some limitations. It focused on health workers’ perspectives and the authenticity of the findings are subjective to participants’ experiences. The study was also limited to pregnant women in Savelugu Municipality. Hence, the findings should be interpreted with caution as they may not be applicable in other settings. Therefore, future studies may consider these limitations in their designs.

## Conclusion

Through a constructivist grounded theory approach, the study found that though a robust referral system exists in the study area, transportation lapses are a drawback to its function. In addition, health workers often fail to accompany referred cases. Findings of this study may be adopted for smooth operation of referral systems in other settings. An inter-sectorial approach to tackling the referral transportation challenges is recommended. Government should develop policies to enhance adequate staffing, and health facility managers should also ensure that all referrals (particularly pregnant women with malaria) are accompanied by a health worker.

## Supporting information

S1 File Minimal dataset. (DOCX)

## Abbreviations

AFRO: African Regional Office
ANC: Antenatal care
CHPS: Community-based Health Planning and Services
EDCTP: European and Developing Countries Clinical Trials Partnership
GHS: Ghana Health Service
OPD: Out-Patient Department
SSA: Sub-Saharan Africa
TDR: Special Programme for Research and Training in Tropical Diseases
WHO: World Health Organization
