# Supplementary material for: Perspectives of health workers on malaria case referral among pregnant women attending antenatal care in Savelugu Municipality, Ghana: A qualitative descriptive study
Source: PLoS One. 2025 Mar 18;20(3):e0319567. doi: 10.1371/journal.pone.0319567 (PMC11918396; doi:10.1371/journal.pone.0319567)
Supplement: S1 File — (DOCX) [file pone.0319567.s001.docx]

| **S/N** | **Background Information of Service Provider** | |
| --- | --- | --- |
|  | **+Questions** | **Response** |
| 1 | What is your position/rank/role? | I am the facility in charge and a senior staff nurse, I consult and prescribe at this facility (**Moglaa RGN**) |
|  |  | I don’t hold any position in this facility. I am ean staff nurse and I render consulting and treatment services to clients (**Moglaa enrolled nurse)** |
|  |  | I am the in charge and a midwifery officer, I supervise my colleagues aside that, I take part in ANCs, take care of Children under five, counsel lactating mothers and mal nutritional cases. (**Moglaa MO**) |
|  |  | I am a staff midwife. I provide antenatal services, post-natal services, delivery services and family planning services. **(Moglaa SMW)** |
|  |  | I am the sub district head and I am a superintendent community health nurse. As a community health nurse I do preventive services; immunization, registrating, ANC, Post natal, CWC, home visits, heath education. **(Savelugu RCH CHN)** |
|  |  | I am a nurse and a senior staff nurse. I see OPD cases, I actually consult and manage cases at the OPD level. I do health education, I also do support with school health and other RCH activities. But my role is the OPD consultations at the OPD level. **(Savelugu RCH, RGN)** |
|  |  | I am the manager here, I am in charge in the facility and the laboratory technician. I have a technologist by the name Dzifa. My role is to supervise because my in charge actually is engaged somewhere so I am the one who have been in charge of supervise (supervision) and make sure the lab is run smoothly here, my director have been also sort of auditing what we run here as well. Because it’s a self-lab from New Life Clinic so my role is to manage the place, manage the staff and the patients as well because the patients are difficult. In the lab I am the one supervise the results, make sure that procedures are been monitored in the lab, make sure the materials are not outdated ones, reagents are fresh and prepared at the appropriate time so that to give quality results. Aside that it is similar to, make sure that patients are well managed, because when they come the complaints come, staff complaints too come, so sometimes outside like Savelugu hospital might have an issue where they need their patients to supervise a case in the lab as well, so sometimes i collaborate with Savelugu hospital and other facilities, so this is more of what we do. **(Mbia Lab, Lab technician)** |
|  |  | I am the Lab manager and a principal medical scientist. To supervise the activities of the lab and to ensure that the lab is always in full service, there is good collaboration between the lab and also the other departments and at the managerial level. **(Savelugu Hospital Lab, Manager)** |
|  |  | I am an enrolled nurse, and a certificate nurse. I am not a midwife but my role here is to help them record what the client will bring if they are out from the examination room, my own is to record what I have seen and put inside the book for them and may be their urine test, that is the urine protein if it is positive or negative that’s what I do**. (Savelugu Hospital, enrolled nurse)** |
|  |  | I am the in charge and a principal midwifery officer. I am a midwife so my role is to take the pregnant women who come under my care and to also make sure that the staff under me are working to expectation and any logistics that they will need for the work I have to make sure it is there for them to work with. **(Savelugu Hospital, PMO)** |
|  |  | I am medical officer in this facility. I am in charge of the maternity unity, emergency, male medical ward, NICU. Sometimes I do attend to patients in the other units. We attend to the pregnant women that have concerns, usually the pregnant women go through ANC some of them come with complaints, where the complaints go beyond the midwives, and then they are referred to us to see. I don’t hold a rank in this facility. My daily routine is to do ward rounds in the units I mentioned and when I am done with my wardrounds, I then attend to the ANC clients who have concerns that are beyond the midwives. And some of them will have issues the need to be admitted, we admit them and manage them in the ward **(Savelugu Hospital, Med. O)** |
|  |  | I am a nurse precisely an enrolled nurse but I’m currently at ANC ward. I work with midwives I directly deal with pregnant women. I deal with newborns and pregnant cases, mostly referral or cases that need immediate attention. Today (Thursday) for instance those who have given birth like yesterday or the day before yesterday and couldn’t come for checkup, so most of them come today we check them to see if the baby and the mother are well, the uterus has returned, breast and other things are okay for the baby and to see if the baby has some complications or the baby is okay **(Savelugu Hospital, enrolled nurse)** |
|  |  | I am a Midwifery officer and a midwife. I take care of pregnant women throughout pregnancy and when in labor and they are sent to the labor ward. Making of requisition and picking supplies from GHMT or the stores, counseling of pregnant women on HIV, then I do family planning, **(Savelugu Hospital, MO)** |
|  |  |  |
| 2 | How long have you been working at this facility? | Two (2) years (**Moglaa OPD, RGN**) |
|  |  | Two years (**Moglaa OPD, enrolled nurse)** |
|  |  | 6 years (**Moglaa RCH, MO**) |
|  |  | 2 months **(Moglaa RCH, SMW)** |
|  |  | About 4 years **(Savelugu RCH CHN)** |
|  |  | I have worked here for the past 6 years. **(Savelugu RCH, RGN)** |
|  |  | Since 2012 (7yrs) **(Mbia Lab, Lab technician)** |
|  |  | I have been here for 9 years **(Savelugu Hospital Lab, Manager)** |
|  |  | I am a newly posted we have just been posted, we are just 3, 4 months. **(Savelugu Hospital, enrolled nurse)** |
|  |  | This my 5th year. **(Savelugu Hospital, PMO)** |
|  |  | I came last year, 2018 May, getting about 1 year 4 months. **(Savelugu Hospital, Med. O)** |
|  |  | I have just been posted, but I have worked here for at least getting to 4 months. **(Savelugu Hospital, enrolled nurse)** |
|  |  | One year **(Savelugu Hospital, MO)** |
|  |  |  |
| **Malaria Case Identification, Management, Referral and Defaulter Tracing** | | |
| 3 | Have you ever received specific training /mentorship on malaria case identification? If yes, when did you receive the specific training/mentorship? | (Training) Yes, 1^st^ in 2016 in Tatale and 2^nd^ May 2019, at Tamale Regional Health Directorate Training Unit.  (Mentorship) Yes, March 2017 in Diare. (**Moglaa OPD, RGN**) |
|  |  | (Training) Yes, In May 2019 at the Regional Health Directorate, Tamale.  (Mentorship) No (**Moglaa OPD, enrolled nurse)** |
|  |  | (Training) Yes, in 2016 at the Savelugu Hospital.  (Mentorship) Yes, In 2016 at the Savelugu Hospital, Dr. Abankwa was my mentor.  **(Moglaa RCH, MO**) |
|  |  | (Training) No, but I have knowledge based the training I had at school. (2013-2016)  (Mentorship) Yes, that was last 2 years (2017) during my National Service at the Ho Municipal Hospital. **(Moglaa RCH, SMW)** |
|  |  | (Training) Yes, but basics. It has been long about 10 years ago at the Savelugu hospital, it was an in-service training.  (Mentorship) No **(Savelugu RCH, CHN)** |
|  |  | (Training) Yes, in 2014 at the Savelugu hospital conference centre.  (Mentorship) Yes, in 2015 and 2017 at the Savelugu District Health Directorate. **(Savelugu RCH, RGN)** |
|  |  | (Training) Yes, that was at Koforidua in 2013, at the Koforidua regional hospital specifically auditorium.  (Mentorship) I think they arranged one in Savelugu here combined with the Staff of the Savelugu hospital, some of us participated actually that was generally voluntary to those who are actually willing, that was when they changed the method of, instead of using the plus 1 method we were advised to use we call it percentage count. That was last year, 2018. At the modern surgical center and another one at New Life Clinic Tamale. **(Mbia Lab, Lab technician)** |
|  |  | (Training) Yes, twice somewhere 2011 and the recent one was in this year, 2019. We were trained to also train some people in the District it was organized by Ghana Health Service under the National Malaria Control Program under the Ghana Health Service in Tamale.  (Mentorship) No. **(Savelugu Hospital Lab, Manager)** |
|  |  | (Training) No, I have not received any training, but I have personal knowledge. When I see you and I know that you may be you are pale the way you are looking may be there is some sign of fever or malaria in you.  (Mentorship) No. **(Savelugu Hospital, enrolled nurse)** |
|  |  | (Training) Yes, about 3 years (2016) ago that one we had it at Tamale Regional Health Directorate, but this year (2019) we had a refresher in Savelugu.  (Mentorship) No **(Savelugu Hospital, PMO)** |
|  |  | (Training) No, but my colleague has received the training she is part of the malaria control program, I don’t know how many times a year, but she has been going for the training, because we are two medical officers in charge of the units, both of us cannot leave the facility at the same time, she is specifically in charge of the malaria training the last training was just about two months ago. I think it was at the Regional Health Directorate or the Tamale Teaching Hospital I am not too sure. I have knowledge from what we were taught at school, and when my colleagues come from the training they also brief us.  (Mentorship) No **(Savelugu Hospital, Med. O)** |
|  |  | (Training) No, but with the training that I have acquired at least they taught me something about midwifery so at least the cases, like a pregnant woman will come to complain to you about the signs and symptoms, they will give you the symptoms and you will also figure out the signs.  (Mentorship) No **(Savelugu Hospital, enrolled nurse)** |
|  |  | (Training) Yes, that was in 2015, I was at Bulsa south district but it was the National Malaria control program that organized it for midwives and we were called in Bolgatanga at the Xtee Crystal Hotel. **(Savelugu Hospital, MO)**  (Mentorship) That was over there, during the SP validation we were taken through how to track the women especially those you have given SP 1 till then you are able to track them to complete the 5^th^ dose of SP. It was a supervisory event. **(Savelugu Hospital, MO)** |
|  |  |  |
| 4 | How is malaria identified among pregnant women attending ANC at your health facility? | 1. For first time clients are given a lab form to go to Savelugu upon return the midwife go through the test results and if Malaria is seen they are treated. 2. RDT test is also done at the facility to determine malaria in pregnant women at this facility. When it is positive, for 1st trimester pregnancy Quinine is given and 2^nd^ and 3^rd^ trimester ACTs are given. (**Moglaa OPD, RGN**) |
|  |  | RDTs are used for malaria identification but in the absence of RDTs the pregnant women are refer them the lab in Savelugu for microscopic test. (**Moglaa OPD, enrolled nurse)** |
|  |  | After counselling and palpation client is asked some questions and based on the response if signs/symptoms (fever, bodily pains etc.) shows malaria we refer them with a note to the OPD where RDT is used to test for malaria. **(Moglaa RCH, MO**) |
|  |  | It depends on the complaints they bring; based on the complaints and signs and symptoms they present, we do the RDT test to identify it but unfortunately we don’t have the RDTs so refer them to the OPD for further management and we use RDT. And also they go to the Lab. Where they do blood film for malaria parasite, so when they come and it’s positive we refer them to go and take medication. That test is done at registration 28 weeks and 35 weeks, so if it’s positive then we continue from there and if it is negative we advise them to sleep under mosquito nets and when they are due for the IPT they take it. **(Moglaa RCH, SMW)** |
|  |  | The clinical signs are there like; fever, vomiting, headaches, chills, loss of appetite and the rest apart form that we refer to the OPD for RDTs to be done. We refer them to the lab but I can’t tell what goes on in the lab. We only send them and they come with the results. **(Savelugu RCH, CHN)** |
|  |  | Mostly when the pregnant women come for ANC the midwives will ask them whether they have any complaints, whether they are sick or not, so when we receive such complaints they do refer them to the OPD. When they come we take their history, make our assessment, do our lab investigations and if the person is positive we manage it accordingly. **(Savelugu RCH, RGN)** |
|  |  | When we have the malaria case we do have 2 methods, we have the one we call the RDT method and the slime method.   1. The RDT method that’s the one we call it, like a carcert, where buffer is provided, and alcohol pad is provided and you prick the dumb of the patient take some sample which you applied on the specific place made for the sample to be applied. The buffer is added by a drop or two. Depending the nature of the sample, when it is anemic you don’t add buffer, so it might run the thing invalid. 2. Slime method, were samples taken are run on the slide and allowed to dry for a few minutes then you stain when you stain you do the normal washing and allow to dry for microscopic examination. So normally basically two methods we use; the RDT and Slime method which is the same as microscopy. **(Mbia Lab, Lab technician)** |
|  |  | We use microscopy and at times RDTs that is if it is available, but most of the time it is microscopy. **(Savelugu Hospital Lab, Manager)** |
|  |  | We will give the person sample to go and check, do the test for us before we now identify, but when we see the person mostly we just look at the person face and know that this person is suffering from either anemic (Anemia) or she is suffering malaria but the right results is that we write a request form for person to go for the test because we don’t do the test. When the person comes and it is positive then we will now declare the person at once. **(Savelugu Hospital, enrolled nurse)** |
|  |  | Here what we do is when they come for the first time we do laboratory test for them and malaria is one of the lab investigation that we collect from them but the subsequent ones we pick it from the signs and symptoms that they will give us and then when they give those signs and symptoms, then we will then ask them to go the clinician and there, they will also assess whatever the woman will bring if they want the woman to do any malaria test then they do and the treatment will go on there. **(Savelugu Hospital, PMO)** |
|  |  | This one, just like any other patient, usually when they come it’s the symptoms and signs we use, if these correlates to malaria infection we let them go to do the lab, sometimes we do the RDT at the OPD level, if it is negative but the woman is still symptomatic then we go ahead and do the blood film. So basically it is the signs and symptoms, the person comes with the symptoms we ask for the signs. **(Savelugu Hospital, Med. O)** |
|  |  | How they normally identify, as I said like Monday to Wednesday pregnant women come for palpitation and we even do some education on like if you are not feeling well, you come we get to identify what’s wrong with you, if it can be treated within the facility the we know how to deal with you, if they come and owns up to tell you, you also get to figure out the signs. Some of the symptoms that we can use to identify in pregnant women include; low immune system, low or some no appetite at all and lower abdominal pains waist pains, general malaise, the lower abdominal pain especially is a factor that you can use to determine malaria in pregnant women. **(Savelugu Hospital, enrolled nurse)** |
|  |  | Every woman picking a card for the first time, you’re tested for malaria among the other routine tests we do, so from there if you’re tested positive then we pick it up from there. When they come again on any other visits and you give complain of may be headache or symptoms of malaria then we let you go and do the test, if it is positive then we will refer you. **(Savelugu Hospital, MO)** |
|  |  |  |
|  |  |  |
| 5 | How are pregnant women who are tested positive with malaria referred from your health facility to the next level of the health system in the past one month (August, 2019)? | The Savelugu Municipality has a referral WhatsApp platform for pregnant women know as the labor room platform with all health workers (Midwives, Medical Assistants, Doctors etc.) on board, where a photo of the client’s referral form is posted on the platform. (**Moglaa OPD, RGN**) |
|  |  | A referral form is filled with client’s details, complaints and the treatment given at this stage. The clients are then accompanied by relatives, but if it is severe the ambulance is used. And a duplicate of the filled referral form is kept in the facility. (**Moglaa OPD, enrolled nurse)** |
|  |  | We fill out the details and complaints of the pregnant woman and the referral facility name in the referral form and post a snapshot on a WhatsApp platform through which midwives at the referral facility are notified. If the condition is severe we use the ambulance. **(Moglaa RCH, MO**) |
|  |  | We have to examine her first and the findings are put in a referral form, we have an ambulance to take them to the hospital. **(Moglaa RCH, SMW)** |
|  |  | We have a referral book, which we fill, after filling we inform the next level that we are bringing a client meanwhile we inform the client that we are referring her, after informing the next level we just give her the referral letter, because we don’t have any means of transport here. **(Savelugu RCH, CHN)** |
|  |  | For the past month we have not referred pregnant woman with malaria cases out of this facility. But when we realize it is complicated malaria, which we cannot manage here we normally refer to the next level which is the Savelugu hospital. So normally we give our pre referral treatment that is injection Arthemeta or injection quinine, after that we get them a referral note, we fill the referral form for them and they will send it to the next level. **(Savelugu RCH, RGN)** |
|  |  | When we identify positive case, normally we use after indicating a result that shows positive, at the back of the results we use a red pen to write at the back malaria positive referred to hospital. Immediately it goes to the midwife, when the midwife see the red pen she knows that it sort of attention so she quickly normally refer them to appropriate doctor who will handle such cases, so that the only way. Because don’t allow them to go home, when they go home we are been even punished, because we are asked to refer any malaria cases not only even malaria cases even low HBs to the hospital and make sure the person is given attention. **(Mbia Lab, Lab technician)** |
|  |  | In the municipality this is the highest, so unless there is a complication, that is when they will be referred outside but if is because of malaria they are normally treated here, unless the person has obstetric com plication or the malaria has brought about some kind of complications that cannot be handled at our level that is when the patient will be transferred, but if it is about malaria they are treated here.  They are transported by an ambulance, arrangement will be made and the relatives will be required to pay some fees, then they will be transported to the teaching hospital, so once they are able to make those things available then they are transported, at times too people have financial challenge but the hospital still transports them, so later they engage the patient relatives and they are able to sort it out. **(Savelugu Hospital Lab, Manager)** |
|  |  | You know this place is not a regional it is a district. So when we get to find out that the case, the treatment that we are supposed to give we don that the equipment here we refer the person.  We make sure that we write a report and put inside the client’s folder then we take another assistant a nurse assistant to assist the patient to the next facility, because we can’t just leave the patient alone, we make sure that we pick one nurse to be part with the relatives of the patient then we refer the person to that place. So that when they reach there the nurse assist will explain why he/she is asked to bring the client. **(Savelugu Hospital, enrolled nurse)** |
|  |  | They have an ambulance and sometimes some go, they have a referral book, they write every detail about the pregnant woman, then where they have treated up to and the drugs they have used. We have a system here they will call if they want to refer to Tamale, they will call Tamale that we are bringing a case for them to know that they are bringing a case, then they will refer and they person will go with the referral note to the next level. **(Savelugu Hospital, PMO)** |
|  |  | As for referral we have a very reboast referral system, the facility we have been able to establish, we have a platform that has midwives, doctors on the platform that when there is any patient that need referral we communicate on the platform, we take the history on the referral sheet we put it on the page, the doctors at the receiving end are on the platform so they will comment. The ambulances we have two that usually convey our patients, so we communicate on phone and on the WhatsApp platform for all our referrals. But for malaria patients personally I haven’t come across any malaria patient that is referred because the person is not doing well, if only its confirmed malaria, the interventions I mentioned earlier they usually work. Unless I go through the report, I can’t think of any malaria patient that was referred because of the malaria. I know with the kids we sometimes refer them, sometimes the person comes with complicated malaria, after some 3 to 5 days of managing if the person is still not coping, those ones we sometimes refer them for the management, but the adults we don’t usually get to that level, for the kids we have referred a number of them on the account of malaria. For the adults we refer them but not because of malaria, if we are referring a person with malaria then maybe the person has other conditions for which we are referring and malaria is part of it. **(Savelugu Hospital, Med. O)** |
|  |  | If you come and we do the test and it is positive we always tell you why we will refer you, we will ask you to go and take a folder, as she tells you the signs you know there is malaria because the test is positive, so if the person tells you the signs and if there is another factor to that you just add up. So when she goes to see the doctor and after everything they will take a midwife or a nurse from the facility to refer the person. I the case is an OPD case they will take the person to OPD, if it is also a maternity case it can be malaria though, but you will need a maternity doctor because in some cases there will be malaria there will be blood lose in the system. So it will be a maternity case, so they will refer to maternity and let the midwife return and tell the doctor or in charge what happened. **(Savelugu Hospital, enrolled nurse)** |
|  |  | The referral form is filled, the client is informed that the situation cannot be handled here, we need to get to a bigger facility where they have specialized people to take care of the person. There is an ambulance that is available, then we get the relatives inform and a nurse accompany the client with the relatives in the ambulance to the next level. At times the next level too is called to inform them about what we are coming with, like let’s say I’m coming with a severe malaria case, this is what we have done and we cannot do this, so we are coming for further management that is the next level we call to inform them. That is basically what we do if we are referring. **(Savelugu Hospital, MO)** |
|  |  |  |
| 6 | Could you describe the referral system of pregnant women with positive malaria? | The relatives of the client are first informed about the referral. Afterwards the referral form is filled and a snapshot is taken and posted on the labor room WhatsApp platform.  A midwife from the referred facility are then alerted by the post and bed preparation is made to receive the client. (**Moglaa OPD, RGN**) |
|  |  | A referral book is filled with client’s details, complaints and treatment provided at this level. The names of both the referral and referred facility is written on the form. Upon acceptance and treatment a feedback form is filled and sent back to the facility. (**Moglaae OPD, Enrolled nurse)** |
|  |  | Pre referral treatment is given and then the referral form is filled out with the client’s details and complaints and the referral facility name and a snapshot of the filled form is posted on a WhatsApp platform. A staff accompanies the pregnant woman, afterwards a feedback form posted on the WhatsApp platform and also a copy is submitted to the facility. **(Moglaa RCH, MO**) |
|  |  | If a client goes to the Lab and the blood film (BF) for malaria parasite is positive, then we write a referral note then you add the results then the ambulance will take the client to the hospital for further management and sometimes based on the signs and symptoms that they present we don’t do any test we just assume its malaria in pregnancy and so we refer them to the hospital for further management. **(Moglaa RCH, SMW)** |
|  |  | Like I just told you, we don’t have any ambulance here. So if it is the simple malaria we treat here but if it is complicated we fill out the referral form for the patient to go to the next level that’s the Savelugu hospital. **(Savelugu RCH, CHN)** |
|  |  | When the come and it is beyond us, we quickly give our pre referral treatment then we fill the referral for them, normally we don’t go with them, unless the person not stable, we support them with our own means, our motorbikes, sometimes we call their relatives if they can support. We do follow up. We have a referral platform in the District such that when you are referring a case you can just quickly give your information. So we do communicate with the next level people. **(Savelugu RCH, RGN)** |
|  |  | The results given like a letter how we staple it we turn at the back we write malaria positive with a red pen, sometimes we only write referred to hospital with a red pen, when it goes to the midwife, so the midwife sees the red pen knows that this is the case that we referred and the results been open will see the number of the malaria, whether it is +1 or +2, it’s the severe one or not severe one that one is been indicated inside the results. But at the back we write malaria positive against the hemoglobin level, so the person is directed to go to the ANC department. **(Mbia Lab, Lab technician)** |
|  |  | The receiving facility will be call and they will find out whether there is a bed and then once they understand themselves then they need to the sign a consent, they fill a referral form and they engage the ambulance service they go through the necessary administrative issues, the nurse accompanies the patient and the relatives and when they get there the nurse accept them and do handing over, they give details as to what has been done at our facility, it’s all documented, so they hand over to the receiving facility and from time to time they call to find out what has happened to the patient and if the patient was successfully treated they give a feedback and when patient passes away they give the feedback. So is like our facility engages the people to know what happened after the referral. **(Savelugu Hospital Lab, Manager)** |
|  |  | We write the person’s history and how pain that is in the person, then after that we do the test even though the person will tell us. After the test and it is positive and the severe one. The in charge or doctor will sign and write a report, the pick a registered nurse or a senior staff member to be part and the person will also write and sign, they will now pick the person to the place. **(Savelugu Hospital, enrolled nurse)** |
|  |  | The referral system here that I know, we have a team and the referral coordinator, everybody knows him. So when you get the case, you first of all inform him that you have a case that you want to refer, so he will the give you the form to fill but some of the specific point we have given the form to them so when it comes yours is to fill, but you have to inform the coordinator, then the coordinator will the next level that they are referring. He also has the referral coordinator’s number, so he will then call that he is bringing a case then they will continue from there. **(Savelugu Hospital, PMO)** |
|  |  | We have referral coordinators in every unit from here and they also have their coordinators (TTH), we have set up coordinators who will communicate, so I don’t necessarily have to call. Once I make it intention clear to the ward, I can just call our coordinator and he call the ward coordinator that the patient is going then they will make arrangement to receive the patient, then they will give us feedback, then we will now tell our ambulance driver. They usually are the in charges, mostly. But a doctor can also call a colleague doctor or we can call the central line at the teaching hospital and they will link it up with the ward line then I will talk to the person, so that’s how we do it.  The WhatsApp platform is not only for referral, it’s for discussion of cases, so if you have a case and have difficulty in managing you can even post it there, somebody can even suggest you refer on the platform, sometimes your intention is not to refer but to seek counsel/medical advice on how to manage a particular client, based on the intervention, a senior colleague can suggest a referral, so in that case you will now refer and post the details on the page. Sometimes you can make the communication before you post on the page, or sometimes you post and make the communication. So there’s fixed regulation on which one comes first. **(Savelugu Hospital, Med. O)** |
|  |  | You bring the folder you do entering the person’s name, gestational age of pregnancy, the person’s age. We find the attendance book if the person was registered here or not we write and if the person is from this town we write the area, if the person is not from this place we also write down, we write every information about the person and why we are referring her.  From here we refer from our unit, so in the other unit too they also have their system of preparation, so if the case cannot be managed they will also have to do the same as we did to refer the person, they call at the unit that they are referring the person to, to alert them that they are referring a patient with this case, so that what we do. **(Savelugu Hospital, enrolled nurse)** |
|  |  | The client is informed that your condition we need a specialized person to take care of you, and we don’t have the facilities and the people to take care of you, so we are taking you to TTH that is the place we will refer you to, with that we will fill the referral form, where you will document every medical attention done to the client on that form, treatment given, vital signs recorded in that form, then client relatives too are informed to prepare so that somebody will accompany the client to the next level, the next level is also called to inform them about you bringing a patient to them. When everything is done the ambulance driver too is informed then you arrange with the relatives, you either wheel the person to the ambulance, then a nurse will accompany to the next level.  When you get there, they will receive, also check whatever you have done, also see to it that the client is alive before taking over from you, so they do those formalities with you before you hand over everything, whatever you have done if they are convinced with what is done on the paper, you will still talk orally, then you hand over the patient and relatives and come back. **(Savelugu Hospital, MO)** |
|  |  |  |
| 7 | How will you describe the functionality of the referral system at your health facility? | It is very functional because there is a referral focal person, there are referral books, feedback forms, register and a means of transport for our referral cases. A midwife accompanies the client. (**Moglaa OPD, RGN**) |
|  |  | It is functional due to the processes we go through in filling out the forms and presence an ambulance and the feedback we receive after the treatment. (**Moglaa OPD, enrolled nurse)** |
|  |  | The referral system is functional, because usually a feedback is given to us which indicates a complete referral case. **(Moglaa RCH, MO**) |
|  |  | It is very good because we have a means for transporting the clients to the hospital. We have the results **(Moglaa RCH, SMW)** |
|  |  | It is functional but I would have been very good if we had a transport system that could send the patient there very early to avoid complications and then may be death. An ambulance or transport system would have made it better to avoid complications. I will rate it 60-70% **(Savelugu RCH, CHN)** |
|  |  | I think it is okay, but the transportation is always a problem if the client is unstable, we call the relatives to get their own transportation. I will rate it 80% **(Savelugu RCH, RGN)** |
|  |  | If I get your question right, I think it is a little difficult in a way it is good. Because when you direct the person without tracing the person might also decide to find her way somewhere, so we point to the place and make sure the person goes, when we refer after some minutes I have to call a midwife if she has seen such a person, when she not there, the facilities are there we find out this person has been tested positive and was advised to see a midwife at Savelugu hospital which actually has not been identified so in case the person comes back check the results, so those at the villages call and say we don’t refer the cases, because we have been accused that we don’t refer, so we have to write with a red pen. So it’s good just that sometimes pregnant women because of some conditions they seek to see their husbands first before attending to such cases sometimes. So the system is good it only need more education, so that they understand the reason why they have to go to the midwife for attention. I will rate it 85%. Only few don’t understand the reason why they are referred **(Mbia Lab, Lab technician)** |
|  |  | I will say it is good, I will rank it as good, because people don’t normally encounter a lot of challenges as I said the major challenge is the financial limitation, some relatives are not able to provide immediate finances for the ambulance to take off but the hospital too has a system to pre finance and then later the relatives are engaged to reimburse. **(Savelugu Hospital Lab, Manager)** |
|  |  | Well if I am to rate it I will give somewhere 80 or 85%. **(Savelugu Hospital, enrolled nurse)** |
|  |  | It is very, very effective is those years that all these things were not in place. But for this time because the system is very active, the coordinators are there. Even we have a platform that even the sub districts when they are also coming to this facility they will also let us know, when we also want to send to another level we also let them know. So is very very effective. **(Savelugu Hospital, PMO)** |
|  |  | So far I think it is working and it should be one of the best. If there any other one I am yet to see, because having a team of experts on the platform like I said it is a multidisciplinary platform where we have people from different backgrounds on the platform, like I said it is not only for referral, sometimes there is something you just have to do and you don’t need to refer again, so if you send the information on the page and then a senior colleague sees it and say this one, you just do this and the problem will be solved. So it has helped a number of patients to seek early treatment. Some of them in the end they don’t even get referred. And also because of the feedback system, so when you send the person the ward receives and give a feedback. And then follow up, once receive the client your management have to be updating the senior colleagues on the page on what you are doing and the outcomes of your interventions. This is done up to the time the patient is stable **(Savelugu Hospital, Med. O)** |
|  |  | I will say it is good, it is functioning well. **(Savelugu Hospital, enrolled nurse)** |
|  |  | It is okay, because since the ambulance is there and the patient is beyond your control, there is nothing you can do than to go, though at times when you get there they give you some answers that you’re not happy with, but I am happy they have received the person and the person is still alive and been cared for. I will rate it 70%. **(Savelugu Hospital, MO)** |
|  |  |  |
| 8 | Do you have additional information on the issues we have discussed so far? | I hope trainings hepatitis B can be organized for us. (**Moglaa OPD, RGN**) |
|  |  | No additional information. (**Moglaa OPD, enrolled nurse)** |
|  |  | We need the RDTs to test these mothers for malaria and hepatitis B in this facility instead of referring them to other facilities for these tests. **(Moglaa RCH, MO**) |
|  |  | No additional information **(Moglaa RCH, SMW)** |
|  |  | No additional information **(Savelugu RCH, CHN)** |
|  |  | No additional information **(Savelugu RCH, RGN)** |
|  |  | Okay, it is good sometimes to inform the pregnant woman for her to know what she is coming to do, you see when you are coming to a place and your sample is taken you don’t know what to do with your sample. It is good to know what tests that they are coming to do so that maybe when you want to give sort of advice it should not be a challenge. **(Mbia Lab, Lab technician)** |
|  |  | No additional information. **(Savelugu Hospital Lab, Manager)** |
|  |  | No additional information. **(Savelugu Hospital, enrolled nurse)** |
|  |  | May be what I will say is, like you are doing research into it, so I will be happy if you will come in with a helping hand, either may be you subsidize the cost of the drug or you give the free. Because of the nature of here, let me say North everybody is poor, the mother is not able to buy the drug, the facility is not able to provide the drug so, as you have come in maybe we hope that, after the research you will come with any package for those mothers. **(Savelugu Hospital, PMO)** |
|  |  | So far the challenge I have seen in my practice is regarding malaria treatment sometimes because of the strict emphasis on test before you treat and also treat only positive cases.  Sometimes the person will come and have the signs and symptoms for some reasons the lab are delaying or maybe the lab can come and it will be negative and they are human elements which can also sometimes affect the results of the lab.  We have had instances where somebody can even be negative and the following day you repeat the lab or send results outside and it will be positive. So because of that sometimes it is usually challenging if you have a patient that you are very sure that this is a positive patient that you want to treat because of the symptoms and it turns out that the lab results is negative, sometimes because of the national policy that we must test the person and treat only positive, usually sometimes affect and most patients are not treated who are actually positive, but because the lab is negative. So i usually have a problem with that and I even confront the pharmacy or the lab, sometimes they also raise those concerns that the insurance policy sometimes only want to pay patients that actually been tested positive, which I think from my experience that should be the way to go, because if you want to put patients life on investigation that also has limitation sometimes we would do great disservice to the patient, even though we are trying to minimize drug abuse or drug resistance but we have to also open the door a bit for some patients instead of just boxing everybody. **(Savelugu Hospital, Med. O)** |
|  |  | Like I said when they come for registration they always let them go and get tested for us to start with any treatment and other things, so with the malaria if you don’t have the malaria parasite in you we give them that goes right, we give some malaria supplements each and every month and it depends on individuals, there are some people who can those drugs and some people the drugs can affect their system and the unborn child, so what we do is that if they get tested and it is negative or even if it is positive they go for management and from here we have our malaria drugs that we give to pregnant women, its mainly for only pregnant women they take it every month for 5 time for prevention of malaria. Because if the mother is not affected the child will not be affected, so we always give those medications till the fifth dose. If the person completes the fith dose it is okay, no malaria to the mother, no malaria to the child. That what we do here.  **(Savelugu Hospital, enrolled nurse)** |
|  |  | No additional information **(Savelugu Hospital, MO)** |
